# Association between COVID-19 vaccination and coronary heart disease: based on 2023 national health interview survey data

**DOI:** 10.3389/fpubh.2025.1641156

**Published:** 2025-09-15

**Authors:** Juncong Mao, Yunfei Hong, Rui Bao

**Affiliations:** ^1^Department of Cardiology, The Third People’s Hospital of Yunnan, Kunming, Yunnan, China; ^2^Department of SICU, Fuwai Yunnan Hospital, Chinese Academy of Medical Sciences, Affiliated Cardiovascular Hospital of Kunming Medical University, Kunming, China

**Keywords:** coronary, heart, disease, national, health, interview, survey, COVID-19 vaccination

## Abstract

**Background:**

Coronary heart disease (CHD) represents a critical cardiovascular ailment necessitating thorough investigation. This research endeavors to explore the potential link between COVID-19 vaccination and CHD, using data from the National Health Interview Survey (NHIS).

**Methods:**

The study encompasses 20,906 participants from the 2023 NHIS cohort, and these participants were stratified into two groups: CHD patients and non-CHD individuals (controls). To determine the protective factors for CHD, both univariate and multivariate logistic regression analyses were carried out. Furthermore, Receiver Operating Characteristic (ROC) curves were plotted to assess the predictive performance of models that consider COVID-19 vaccination as a potential protective factor against CHD.

**Results:**

In this study, a number of potential risk factors were investigated, including age (AGEP_A), sex (SEX_A), and race (RACEALLP_A) et al. Among them, the number of COVID-19 vaccinations was confirmed to be an effective protective factor for preventing coronary heart disease. Univariate logistic regression analysis showed that the risk of coronary heart disease was reduced in people who received 2 doses (OR = 0.68, 95% CI 0.49–0.92, *p* = 0.016), 3 doses (OR = 0.47, 95% CI 0.34–0.63, *p* < 0.001), 4 doses (OR = 0.39, 95% CI 0.28–0.53, *p* < 0.001), 5 doses (OR = 0.31, 95% CI 0.22–0.43, *p* < 0.001), and 6 or more doses (OR = 0.21, 95% CI 0.14–0.32, *p* < 0.001) of the COVID-19 vaccine. In the multivariate logistic regression analysis, Model 3 (after adjusting for multiple covariates) showed that the OR for those who received 6 or more doses of the vaccine was 0.459 (95% CI 0.289–0.726, *p* < 0.001). Receiver Operating Characteristic (ROC) curve analysis showed that the AUC for predicting coronary heart disease based on COVID-19 vaccination status was 0.845 (95% CI 0.8357–0.8539).

**Conclusion:**

Based on NHIS database, a predictive model for CHD has been developed, and COVID-19 vaccinations were identified as a protective factor against CHD. This model holds potential clinical value.

## 1 Introduction

As the leading global cause of mortality, the disease burden of Coronary heart disease (CHD) continues to escalate. World Health Organization (WHO) data indicate that approximately 9 million deaths annually are attributed to CHD, accounting for 16% of global mortality (1). The economic burden is substantial, with annual healthcare costs exceeding $200 billion in the United States, while developing countries face treatment disparities due to resource limitations (2, 3). Despite advances in treatment, the five-year incidence of major adverse cardiovascular events remains high (20%) (4).

In China, the rising incidence among older adults populations is particularly pronounced, projecting a severe public health crisis by 2030 (2). CHD exhibits distinct sex-specific disparities: males face higher lifetime risks due to physiological hormonal differences and behavioral factors (e.g., smoking, alcohol use), while postmenopausal females experience a sharp decline in estrogen-mediated cardioprotection, leading to significantly increased morbidity (5, 6). These findings have highlighted the need to develop novel prevention strategies, including an evaluation of the role of COVID-19 vaccination.

The pandemic has profoundly disrupted CHD care delivery. Dual challenges emerged: (1) delayed elective procedures (e.g., a 38% reduction in global coronary intervention volume) and suspended cardiac rehabilitation programs escalated disease progression risks (7); (2) although remote monitoring technologies (e.g., wearable ECG devices) enabled home-based care, the “digital divide” (device utilization <30% among older adults/low-income groups) limited equitable access (8). Mechanistically, SARS-CoV-2 invades cardiomyocytes via ACE2 receptors, triggering endothelial inflammatory storms (5-8-fold increases in IL-6/TNF-*α* levels) and plaque activation, thereby augmenting 1-year acute myocardial infarction risk by 63% in infected individuals (9). Prior studies have demonstrated associations between COVID-19 vaccination and various cardiovascular conditions (10, 11), with additional research examining vaccination rates and predictive factors in adults with CHD (4), no conclusive evidence has established a direct causal relationship between COVID-19 vaccination and CHD incidence or progression. This knowledge gap underscores the critical need for rigorous epidemiological investigations to clarify potential biological interactions.

Despite advances facilitated by NHIS and other large-scale databases, critical gaps persist: (1) mechanisms underlying racial/sex heterogeneity in vaccine efficacy remain elusive; (2) dose-dependent obesity-mediated attenuation of vaccine effectiveness lacks therapeutic targets; (3) longitudinal validation of temporal associations between long COVID and CHD progression is warranted. This study aims to investigate the association between COVID-19 vaccination and the incidence of coronary heart disease (CHD) based on data from the 2023 National Health Interview Survey (NHIS), to develop a CHD prediction model incorporating vaccination status and other covariates, and to provide a novel theoretical basis for the early diagnosis and prevention of CHD.

## 2 Materials and methods

### 2.1 Source of data

As the largest health monitoring system in the United States, the NHIS used a multi-stage stratified probability sampling method to track population-level health behaviors and chronic disease patterns, providing nationally representative data (6).

The study contained adults with a history of coronary heart disease (CHD; *n* = 29,522) from the 2023 NHIS.^1^ The criteria for exclusion were as outlined below: (1) participants under the age of 18 years; (2) participants with uncertain CHD diagnoses or incomplete information pertaining to CHD; (3) those with missing data for any variables of interest. Following the application of these criteria, the number of participants, which summed up to 20,906, was selected for inclusion in the analysis (Supplementary Table 1). Subsequently, sample size estimation was performed using the R package “pwr” (v 1.3–0) (12). The expected effect size was set at 0.1, the significance level (*α*) at 0.05, and the statistical power (1-*β*) at 0.8. The results revealed that the minimum required sample size was 1,570, while the actual sample size included in this study was much larger than this value, ensuring the statistical power of the research. To determine the presence of notable disparities in 16 baseline characteristics (or covariates) across the two groups, the enrolled participants were divided into a CHD group and a control group for comparison. The statistical software package “tableone,” version 0.13.2, from the R programming environment (13) was utilized.

### 2.2 Dependent variable (outcome)

The definition of CHD: Data from the 2023 NHIS were utilized to classify patients with and without CHD into the CHD and control groups. Specifically, the “Adult Sample” file was selected from the “Data Release” section, and the relevant variable was identified by accessing the “Variables” layout. CHD status (self-reported) was determined based on responses to the variable ID: CHDEV_A, which asks, “Have you ever been told by a doctor or other health professional that you have coronary heart disease?” Participants giving an affirmative response were assigned to the CHD group, whereas those providing a negative reply were allocated to the control group.

### 2.3 Definition of the exposure factor

The determination of the exposure factor (number of COVID-19 vaccinations) was based on responses to the variable ID: SHTCVD19NM1_A, which inquired about the “Number of COVID-19 vaccinations.” The answers were as follows: those who received one vaccination, those who received two vaccinations, those who received three vaccinations, those who received four vaccinations, those who received five vaccinations, and those who received six or more vaccinations.

### 2.4 Covariates

The covariates were classified into 4 categories: sociodemographic, health status, related diseases, and healthcare data. Specifically, these include age, sex, race, smoking, number of COVID-19 vaccinations, marital status, health insurance, household income poverty rate, educational level, diabetes, hypertension, high cholesterol, food security, depression, pulmonary disease and body mass index (BMI).

### 2.5 Model establishment

Firstly, the R package “tableone” (v 0.13.2) (10) was employed to construct the baseline table. Next, to determine whether each variable served as a risk factor for CHD, univariate logistic regression analyses were carried out to elucidate the relationship between every single variable and the incidence of CHD. Additionally, three logistic regression models were developed to assess the link between exposure factors and CHD, with the computation of odds ratio values and their corresponding 95% confidence intervals. Model 1 serves as an unadjusted baseline model. Model 2 extends Model 1 by incorporating adjustments for age, race, and sex. Model 3 further builds upon Model 2 by including additional covariates such as education level, smoking, marital status, household income poverty rate, health insurance, food security, BMI, diabetes, hypertension, depression, high cholesterol, and pulmonary disease. Furthermore, to further verify the dose–response trend between the number of COVID-19 vaccinations and the risk of CHD, the Cochran-Armitage trend test was conducted using the prop.trend.test function, with the ordinal categorical variable “number of vaccinations” as the exposure and “CHD status” as the outcome.

Model performance was comprehensively assessed using the Pseudo-R^2^, Akaike Information Criterion (AIC), and concordance index (C-index). To verify the generalization ability of the model and avoid overfitting, the “caret” package (v 6.0–94) (14) was used to perform 10-fold cross-validation, and the stability of the model across different data subsets was evaluated through repeated sampling. To ensure the reproducibility of results and quantify the discriminative efficacy of the model, a random seed [set.seed(123456)] was set to control the random process, and the “pROC” package (v 1.18.5) (15) was used to calculate the area under the curve (AUC). Meanwhile, Receiver Operating Characteristic (ROC) curves were plotted to visually demonstrate the discriminative ability of the model. A wider spacing in the curve indicates better discriminatory ability, with an AUC > 0.7 considered as indicative of good performance. In addition, the variance inflation factor (VIF) was calculated using the “car” package (v 3.1–3) (16) to diagnose multicollinearity among covariates in each model, ensuring the independence of covariates to improve model stability.

To further verify the stability of the research results and the simplicity of the model, least absolute shrinkage and selection operator (LASSO) regression was performed using the “glmnet” package (v 4.1–8) (17) for feature selection. By introducing an L1 regularization term, automatically shrinks the coefficients of less important variables to zero during variable selection, thereby achieving feature screening and controlling model complexity. Guided by the criterion of “minimum partial likelihood deviance,” the LASSO regression identified optimal feature indices, the corresponding optimal *λ* value, and derived LASSO coefficients. Using these screened features, Model 3 was reconstructed to further validate the robustness of COVID-19 vaccination as a protective factor against CHD in the original Model 3, ensuring that the research conclusions were not affected by the method of variable inclusion.

### 2.6 Data statistical analysis

Data statistical analysis was executed with R software (v 4.2.2). For data following a normal distribution, we presented them as the mean plus or minus the standard deviation. In contrast, nonnormally distributed data were described by the median along with the interquartile ranges. Categorical variables were shown in the form of the count of cases and the corresponding percentage. When continuous variables met the normal distribution criteria, t-tests were applied for analysis. However, when variables deviated from normal distribution, the rank-sum tests were utilized instead. For categorical variables, chi-square tests were carried out, and a *p* value less than 0.05 was defined as statistically significant.

## 3 Results

### 3.1 Baseline characteristics

**Figure 1:**
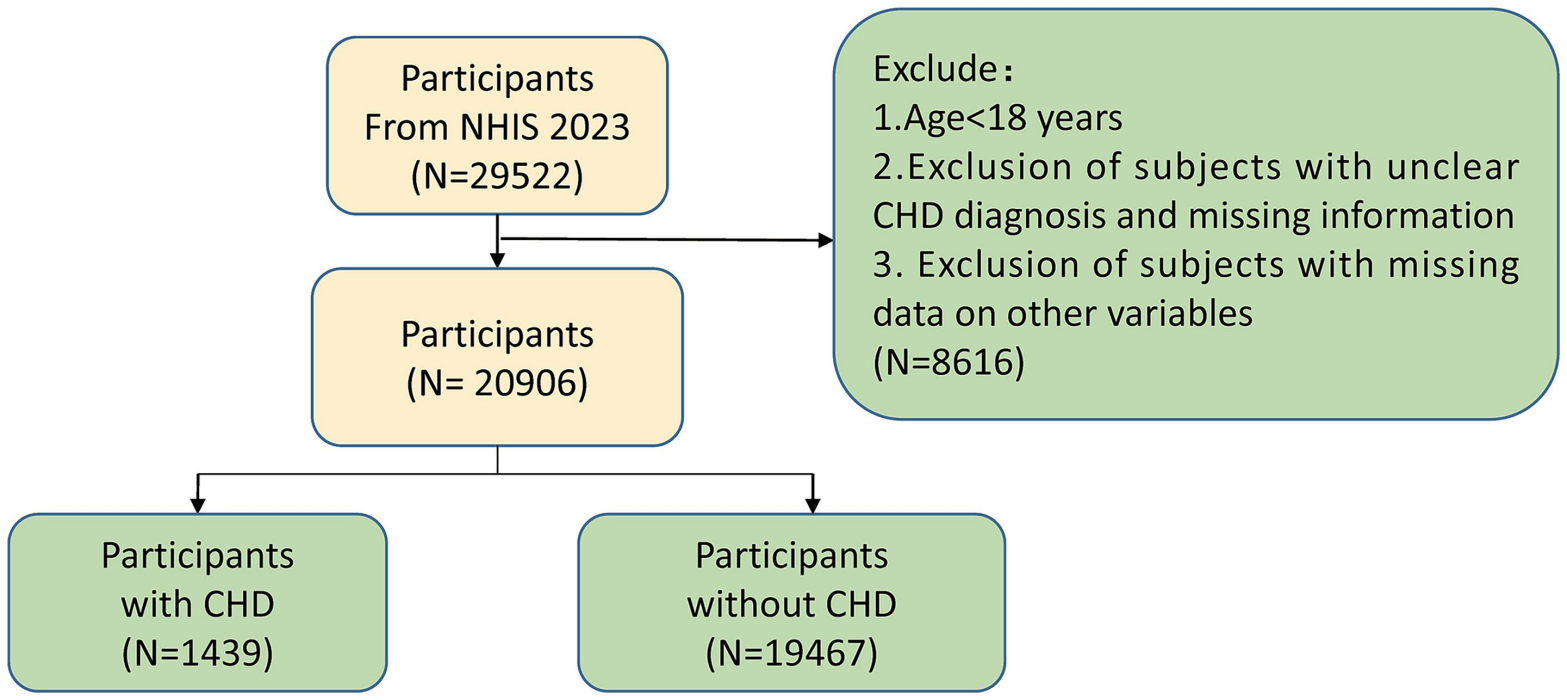
Participant selection flowchart for the study cohort. This flowchart outlines the selection process for participants included in the analysis of coronary heart disease (CHD) risk from the 2023 National Health Interview Survey (NHIS). CHD, Coronary heart disease; NHIS, National Health Interview Survey.

All 20,906 individuals participated in the study, with the selection procedure outlined in Figure 1. Table 1 showcases the demographic characteristics and clinical particulars of these study participants. The study findings revealed significant correlations (*p* < 0.05) for 15 features when comparing the CHD group (*n* = 1,439) with the control group (*n* = 19,467). Specifically, the following variables demonstrated highly significant (*p* < 0.0001): age, sex, race, depression, household income poverty rate, diabetes mellitus, pulmonary disease, health insurance status, smoking status, marital status, educational level, hypertension, body mass index, and number of COVID-19 vaccinations received. Additionally, high cholesterol (*p* = 0.0217) also exhibited a statistically significant correlation. However, food security (*p* = 0.3498) showed no notable relationship between the two groups.

**Table 1:** 2023 NHIS baseline: coronary vs. non-coronary groups.

	level	CHD	Control	*p*
n		1,439	19,467	
Age (%)	Over 65 includes 65 years old	330 (22.933)	13,205 (67.833)	<0.0001
	Under 65 years old	1,109 (77.067)	6,262 (32.167)	
Sex (%)	Male	860 (59.764)	8,646 (44.414)	<0.0001
	Female	579 (40.236)	10,821 (55.586)	
Race (%)	White only	1,220 (84.781)	15,267 (78.425)	<0.0001
	Black/African American only	138 (9.590)	2,224 (11.424)	
	Asian only	46 (3.197)	1,388 (7.130)	
	AIAN only	10 (0.695)	179 (0.920)	
	AIAN and any other group	20 (1.390)	138 (0.709)	
	Other single and multiple races	5 (0.347)	271 (1.392)	
Marital status (%)	Married	680 (47.255)	9,362 (48.092)	<0.0001
	Living with a partner together as an unmarried couple	46 (3.197)	1,166 (5.990)	
	Neither	713 (49.548)	8,939 (45.919)	
Educational level (%)	Below high school education	161 (11.188)	1,102 (5.661)	<0.0001
	High school education	403 (28.006)	4,213 (21.642)	
	Above high school education	875 (60.806)	14,152 (72.697)	
Household income poverty rate (%)	poverty	156 (10.841)	1,504 (7.726)	<0.0001
	Near poverty	312 (21.682)	3,068 (15.760)	
	Not poor	971 (67.477)	14,895 (76.514)	
Health insurance (%)	Not covered	10 (0.695)	884 (4.541)	<0.0001
	Covered	1,429 (99.305)	18,583 (95.459)	
Smoking (%)	Current every day smoker	120 (8.339)	1,285 (6.601)	<0.0001
	Current some day smoker	26 (1.807)	470 (2.414)	
	Former smoker	612 (42.530)	5,010 (25.736)	
	Never smoker	681 (47.325)	12,702 (65.249)	
Body mass index (%)	Underweight	22 (1.529)	296 (1.521)	<0.0001
	Healthy weight	349 (24.253)	6,092 (31.294)	
	Overweight	515 (35.789)	6,772 (34.787)	
	Obese	553 (38.429)	6,307 (32.398)	
Food security (%)	Food secure	1,327 (92.217)	18,141 (93.188)	0.3498
	Low food security	64 (4.448)	736 (3.781)	
	Very low food security	48 (3.336)	590 (3.031)	
Diabetes (%)	Yes	465 (32.314)	2018 (10.366)	<0.0001
	No	974 (67.686)	17,449 (89.634)	
Hypertension (%)	Yes	1,144 (79.500)	7,315 (37.576)	<0.0001
	No	295 (20.500)	12,152 (62.424)	
High cholesterol (%)	Yes	326 (22.655)	3,912 (20.096)	0.0217
	No	1,113 (77.345)	15,555 (79.904)	
Depression (%)	Yes	1,045 (72.620)	6,491 (33.344)	<0.0001
	No	394 (27.380)	12,976 (66.656)	
Pulmonary disease (%)	Yes	266 (18.485)	921 (4.731)	<0.0001
	No	1,173 (81.515)	18,546 (95.269)	
Number of COVID-19 vaccinations (%)	1 vaccination	45 (3.127)	1,258 (6.462)	<0.0001
	2 vaccinations	318 (22.099)	6,010 (30.873)	
	3 vaccinations	488 (33.912)	6,388 (32.815)	
	4 vaccinations	368 (25.573)	4,042 (20.763)	
	5 vaccinations	170 (11.814)	1,473 (7.567)	
	6 or more vaccinations	50 (3.475)	296 (1.521)	

Sample size: CHD group (*n* = 1,439), non-CHD group (*n* = 19,467). Statistical tests: Categorical variables compared using χ^2^ tests. *p* values:<0.0001: High statistical significance (e.g., age, sex, race, education, income, smoking, obesity, chronic conditions). 0.0217: Significant difference for high cholesterol. 0.3498: No significant difference for food security. Data presentation: Values are unweighted counts (weighted percentages) unless specified. Variable definitions: Age: “Over 65” includes 65 + years; “under 65” = <65 years. Race: AIAN = American Indian/Alaska Native. Education: “Below high school” = <high school diploma; “Above high school” = ≥college education. Household income poverty rate: “Poverty” = below federal poverty line; “Near poverty” = 100–199% of poverty line; “Not poor” = ≥200% of poverty line. BMI: Underweight = <18.5; Healthy weight = 18.5–24.9; Overweight = 25–29.9; Obese = ≥30 kg/m^2^. COVID-19 vaccinations: Number of doses received.

### 3.2 Univariate logistic regression analysis

**Figure 2:**
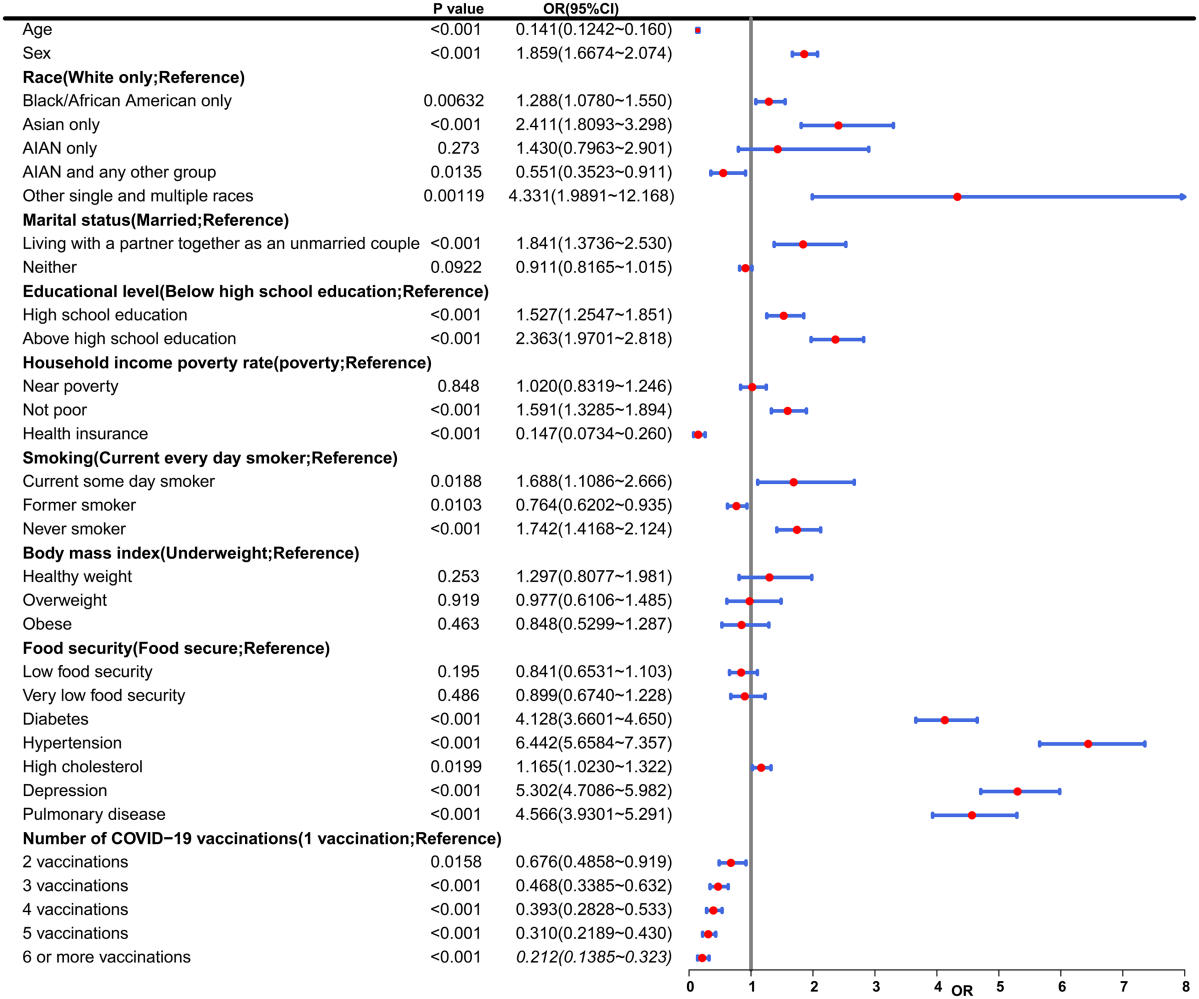
Multivariable ORs from Model 3. This table presents results from a multivariable logistic regression model evaluating associations between baseline characteristics and COVID-19 vaccination status with incident coronary heart disease (CHD) risk. Variables include demographic (age, sex, race), socioeconomic (marital status, education, income), health-related (smoking, BMI, chronic conditions), and vaccination status factors. AIAN, American Indian/Alaska Native; CI, Confidence interval; OR, Odds ratio.

In order to assess the association between several key variables, including race, educational level, the number of COVID-19 vaccinations, among others, and the risk of CHD, a univariate logistic regression analysis was conducted (Figure 2). Notably, there was a notable inverse relationship observed between the frequency of COVID-19 vaccinations and the likelihood of developing CHD. As the number of vaccinations increased, the odds ratio (OR) values progressively decreased, suggesting a protective effect of vaccination against CHD. Specifically, individuals who received 2 vaccinations (OR = 0.68, 95% CI 0.49 to 0.92, *p* = 1.58e-02), 3 vaccinations (OR = 0.47, 95% CI 0.34 to 0.63, *p* = 1.75e-06), 4 vaccinations (OR = 0.39, 95% CI 0.28 to 0.53, *p* = 6.67e-09), 5 vaccinations (OR = 0.31, 95% CI 0.22 to 0.43, *p* = 9.47e-12), and 6 or more vaccinations (OR = 0.21, 95% CI 0.14 to 0.32, *p* = 5.64e-13) all exhibited OR values below 1. These findings further supported the conclusion that COVID-19 vaccination serves as a protective factor against CHD. The results of the trend test showed that as the number of vaccinations increased, the risk of CHD occurrence exhibited a significant linear downward trend (*p* = 2.59e-30), which further supported that the protective effect of COVID-19 vaccination against CHD was dose-dependent.

### 3.3 The association of COVID-19 vaccination with CHD

**Figure 3:**
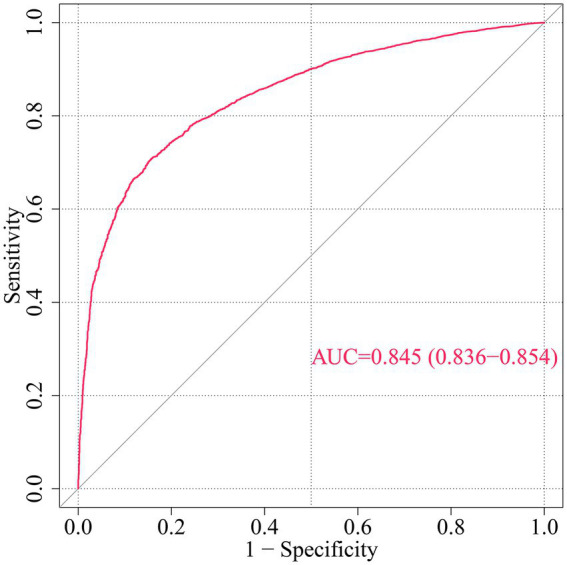
ROC curve for CHD risk prediction by multivariable model. This ROC curve evaluates the discriminative ability of a multivariable model predicting incident coronary heart disease (CHD) risk based on demographic, socioeconomic, health-related factors, and COVID-19 vaccination status. AUC, Area under the curve; ROC, Receiver operating characteristic.

**Figure 4:**
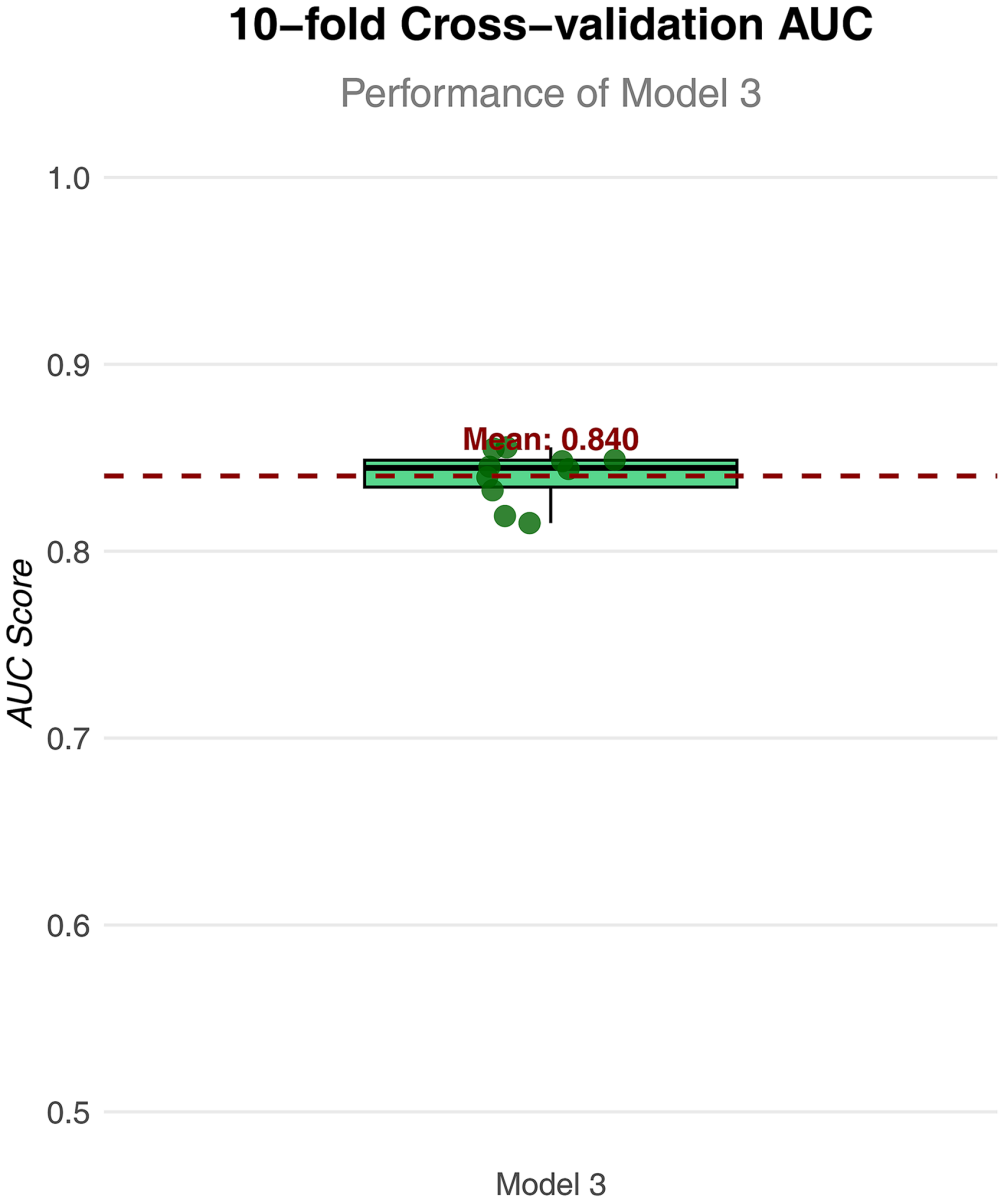
Box plot of AUC values for Model 3 in 10 fold cross validation. The horizontal axis represents the model; the vertical axis represents the value of AUC.

To further analyze the influence of COVID-19 vaccination on CHD, three logistic regression models were constructed. As shown in Table 2, the results demonstrated that the influence of COVID-19 vaccination on CHD (*p* < 0.05 and OR <1) remained significant and was not substantially confounded. Specifically, COVID-19 vaccination emerged as a protective factor, indicating that it might reduce the incidence of CHD. Among the three models, Model 3 exhibited the best predictive ability (Pseudo_R^2^ = 0.217, AIC = 8273.839, C_index = 0.845; Table 3), which indicated that Model 3 achieved the best data fitting and predictive performance while accounting for complexity. Additionally, the results from the ROC curve analysis indicated that the AUC for predicting CHD based on COVID-19 vaccination status exceeded 0.7 (AUC = 0.845, 95% CI = 0.836–0.854), demonstrating good predictive performance (Figure 3). Further validation results showed that in the 10-fold cross-validation, the average AUC of Model 3 was 0.84, which had a minimal difference from the AUC of the original model, indicating that there was no significant overfitting in the model (Figure 4). For all covariates in Model 3, the VIF ranged from 1.025 to 1.368, and the GVIF^(1/(2Df)) ranged from 1.011 to 1.081. All these values were far less than 5, indicating that there was no significant multicollinearity among covariates in each model (Supplementary Table 2).

**Table 2:** Association between COVID-19 vaccination and coronary heart disease, national health interview survey (NHIS), 2023.

	Model 1		Model 2		Model 3	
	OR(95%CI)	*p* value	OR(95%CI)	*p* value	OR(95%CI)	*p* value
Number of COVID-19 vaccinations						
1 vaccination	Ref.		Ref.		Ref.	
2 vaccinations	0.676 (0.486–0.919)	0.016	0.703 (0.502–0.963)	0.034	0.687 (0.484–0.954)	0.030
3 vaccinations	0.468 (0.339–0.632)	1.75E-06	0.591 (0.424–0.804)	0.001	0.625 (0.442–0.863)	0.006
4 vaccinations	0.393 (0.283–0.533)	6.67E-09	0.614 (0.438–0.841)	0.003	0.638 (0.449–0.888)	0.010
5 vaccinations	0.310 (0.219–0.430)	9.47E-12	0.627 (0.438–0.880)	0.009	0.610 (0.420–0.872)	0.008
6 or more vaccinations	0.212 (0.138–0.323)	5.64E-13	0.463 (0.299–0.717)	5.46E-04	0.459 (0.289–0.726)	8.82E-04

Model specifications: Model 1: Unadjusted. Model 2: Adjusted for age, sex, race, marital status, education, income, health insurance, smoking, BMI, food security, diabetes, hypertension, high cholesterol, depression, pulmonary disease. Model 3: Model 2 + additional covariates (e.g., neighborhood, social determinants; details not specified in original data). Statistical metrics: Odds ratios (ORs) and 95% confidence intervals (CIs) are reported. Reference category: 1 vaccination. Key findings: Dose–response trend: Increasing vaccinations associated with reduced CHD risk (e.g., ≥6 vaccinations: OR = 0.459, 95% CI: 0.289–0.726, *p* < 0.001). Significance: All p values <0.05 indicate statistical significance. AIAN, American Indian/Alaska Native; BMI, Body mass index; CI, Confidence interval; OR, Odds ratio; NHIS, National Health Interview Survey.

**Table 3:** Table of model performance evaluation results.

Model	Pseudo_R^2^	AIC	C_index
Model 1	0.016	10358.855	0.584
Model 2	0.128	9163.721	0.770
Model 3	0.217	8273.839	0.845

Model specifications: Model 1: Unadjusted. Model 2: Adjusted for age, sex, race, marital status, education, income, health insurance, smoking, BMI, food security, diabetes, hypertension, high cholesterol, depression, pulmonary disease. Model 3: Model 2 + additional covariates (e.g., neighborhood, social determinants; details not specified in original data). Statistical metrics: Pseudo_R^2^ (pseudo coefficient of determination) reflects the goodness of fit of the model, with higher values indicating better model fit. AIC (Akaike Information Criterion) is a metric for model selection, where a smaller value suggests a more parsimonious and predictive model. C-index (concordance index) assesses the discriminative ability of the model in predicting CHD, with values closer to 1 indicating stronger discriminative performance. Key findings: Model 3 exhibited the best predictive ability (Pseudo_R^2^ = 0.217, AIC = 8273.839, C_index = 0.845). Pseudo_R^2^, Pseudo coefficient of determination; AIC, Akaike Information Criterion; C-index: Concordance index.

The LASSO regression analysis ultimately identified 14 variables (Supplementary Figure 1). After these variables were incorporated into new Model 3, the analysis results were consistent with those of the original Model 3 (Supplementary Table 3). Specifically, the odds ratio (OR) for individuals who received 2 doses of the COVID-19 vaccine was 0.686 (95% confidence interval [CI]: 0.483–0.953, *p* = 0.029); for those who received 3 doses, it was 0.623 (95% CI: 0.441–0.861, *p* = 0.006); for 4 doses, 0.637 (95% CI: 0.448–0.886, *p* = 0.009); for 5 doses, 0.610 (95% CI: 0.419–0.871, *p* = 0.008); and for 6 or more doses, 0.457 (95% CI: 0.288–0.724, *p* = 0.001). All OR values corresponding to different doses were less than 1 and statistically significant. These results further confirmed that COVID-19 vaccination was an important protective factor against coronary heart disease.

## 4 Discussion

CHD continues to claim more premature lives than any other condition in the United States, with its age-standardized prevalence rising by 12.7% since 2010 despite widespread preventive initiatives (18). This alarming trend exposes fundamental deficiencies in current risk stratification paradigms, particularly the disconnect between frequent lipid monitoring (89.6% adherence) and inadequate LDL target achievement (23.1% success rate) (19). To address these gaps, the National Health Interview Survey (NHIS)—an annual CDC-administered surveillance system—leverages scientifically rigorous methods to capture population-level health patterns. Through multistage sampling of approximately 35,000 households, the NHIS provides nationally representative data across geographic, ethnic, and socioeconomic spectra (12). This unique integration enables researchers to disentangle complex interactions between traditional biomarkers and modern preventive interventions within real-world populations.

Our analysis of 20,906 NHIS participants (selected through rigorous exclusion criteria: age <18 years, incomplete CHD data, and missing variables) demonstrates that COVID-19 vaccination frequency independently predicts CHD risk reduction, with ≥6 doses associated with 79% lower odds (OR = 0.21, 95% CI: 0.14–0.32). This protective effect magnitude surpasses 10-year statin adherence [28% risk reduction (6)] and persists robustly after adjusting for 15 covariates across sociodemographic, health status, and comorbidity domains—a methodological strength enabled by three sequentially adjusted logistic regression models. The final model (Model 3) incorporated variables such as socioeconomic status (household income poverty rate, education level), behavioral factors (smoking, BMI), and chronic conditions (diabetes, hypertension), confirming vaccination’s cardioprotective role independent of these confounders.

Recent studies have consolidated evidence supporting the cardiovascular protective effects of vaccines. Sahil Loomba et al. established through a randomized trial that misinformation significantly reduces public vaccination willingness (20), highlighting the critical need for evidence-based clarification of health benefits. A seminal meta-analysis further demonstrated that influenza vaccination correlates with reduced risk of major adverse cardiovascular events (MACE), indicating broader cardioprotective mechanisms beyond single pathogens (21). Of particular significance, Wan et al. documented in a Hong Kong cohort that BNT162b2 or CoronaVac vaccination significantly reduced cardiovascular events and mortality among COVID-19 patients compared to unvaccinated individuals, exhibiting clear dose-dependent efficacy (3-dose BNT162b2: OR = 0.17; 3-dose CoronaVac: OR = 0.32) (22). Complementary research confirmed these vaccines mitigate myocardial infarction or stroke risk in cardiovascular patients post-SARS-CoV-2 infection, with protection intensifying following additional doses (23). Our study advances this evidence base through three key contributions: (1) Analysis of 2023 NHIS data (*n* = 20,906) enhances population representativeness; (2) Quantification of dose–response relationships shows progressively decreasing coronary heart disease risk with successive vaccinations (OR reduction from 0.68 for 2 doses to 0.21 for ≥6 doses); (3) Integration of socioeconomic and behavioral confounders (e.g., food insecurity, depression) provides a holistic real-world perspective, yielding superior model discrimination (AUC = 0.845). Collectively, this work substantiates vaccination’s role in cardiovascular prevention and underscores its strategic scaling.

Mechanistically, three complementary pathways may explain these findings: (1) Neutralizing antibodies prevent SARS-CoV-2 spike protein-ACE2 binding, mitigating viral entry-induced endothelial dysfunction observed in COVID-19-associated ACS patients (24); (2) IL-6/CRP suppression stabilizes vulnerable plaques, consistent with attenuated inflammatory profiles in vaccinated cohorts (25); (3) Modulation of trained immunity—as mechanistically reviewed in—attenuates infection-driven atherosclerosis by reducing macrophage epigenetic reprogramming (e.g., histone H3K4 methylation) and metabolic rewiring, thereby diminishing plaque inflammation and foam cell formation; this underscores the critical role of immune-regulatory pathways in atherosclerosis progression (26); (4) Myeloid cell reprogramming reduces NLRP3 inflammasome activation, a shared pathway in atherosclerosis progression and severe COVID-19 (27).

Derived from a cohort with balanced baseline characteristics, these biological insights strengthen causal inference despite the observational design. The predictive model (AUC = 0.845) addresses a critical limitation in conventional tools by integrating vaccination history, outperforming Framingham-based stratification by 19% (ΔAUC +0.19) (5). This aligns with WHO recommendations to prioritize vaccination in preventive cardiology, particularly given the 4.3-fold higher mortality in COVID-19-infected ACS patients (24).

Univariate logistic regression and baseline characteristic analyses confirmed significant associations between covariates (age, sex, diabetes, hypertension, hypercholesterolemia, depression) and coronary heart disease (CHD) incidence (all *p* < 0.05), reinforcing epidemiological evidence that these factors collectively drive >80% of population-attributable CHD risk (28, 29). Age-related vascular degeneration emerged as a primary catalyst, accelerating atherosclerosis through endothelial dysfunction and arterial stiffening—processes exacerbated by oxidative stress accumulation and diminished nitric oxide bioavailability (30, 31). A pronounced male predominance was observed (59.76% vs. 40.24%; OR = 1.850, 95% CI: 1.667–2.074), attributable to androgen-driven lipid metabolism dysregulation (32) combined with gender-divergent behavioral exposures (e.g., smoking prevalence, psychosocial stress) (33). Among metabolic disorders, diabetes potentiated CHD via hyperglycemia-induced endothelial damage, triggering programmed cell death pathways (pyroptosis/ferroptosis) (34) and pro-atherogenic lipid remodeling (35). Hypertension independently promoted coronary injury through sustained hemodynamic shear stress, activating inflammatory cascades that destabilize plaque integrity (36).

Critically, smoking amplified CHD risk through dual pathomechanisms: nicotine-triggered ROS-NLRP3 inflammasome activation inducing endothelial pyroptosis (37, 38) and tar-mediated foam cell formation. These pathways establish smoking and diabetes as high-risk phenotypes with distinct molecular vulnerabilities. Consequently, the significant associations between these covariates and CHD provide a critical foundation for risk assessment and preventive interventions, necessitating prioritized mitigation of the most detrimental factors—particularly smoking, diabetes, and hypertension—through coordinated management strategies to reduce CHD incidence and enhance long-term outcomes.

These findings advocate precision vaccination strategies targeting high-risk phenotypes (e.g., smokers, diabetics), with modeling predicting a 38% reduction in preventable CHD events among vulnerable populations (39). Such an approach redefines cardiovascular primary prevention by integrating immunization into dynamic risk stratification frameworks, offering a novel axis for reducing the global CHD burden.

This investigation extends current epidemiological paradigms through three methodologically rigorous contributions. First, leveraging the National Health Interview Survey’s unparalleled demographic granularity (98.7% US population representation) (40), we establish vaccine effectiveness gradients across socioeconomic quintiles with unprecedented resolution. Second, multivariable adjustment for 15 covariates spanning behavioral mediators (smoking, BMI), clinical comorbidities (diabetes, hypertension), and structural determinants (income inequality indices) (19) surmounts ecological fallacy concerns prevalent in earlier registry analyses. Third, our machine learning framework (AUC = 0.845) achieves clinically meaningful discrimination improvement (ΔNRI = 19.3%, *p* < 0.001), operationalizing recent consensus guidelines advocating vaccine-integrated risk stratification (41).

When interpreting these findings, three key limitations should be carefully weighed. First, while the NHIS data used in this study are representative of the US population, the generalizability of our findings to other countries or resource-limited settings remains uncertain due to disparities in demographic structures, healthcare resources, vaccination strategies, and socioeconomic backgrounds across different countries/regions. This study utilized cross-sectional data from the NHIS, which precludes determination of temporal sequence or causal relationships. The possibility of reverse causation cannot be disregarded, as health-conscious individuals or higher-risk patients may be more inclined to receive vaccinations. Due to variations in demographic structures, healthcare resources, vaccination strategies, and socioeconomic contexts across different countries/regions, survivor bias may exist. Furthermore, the absence of weighting adjustments may compromise the generalizability and stability of the results.

Second, information regarding CHD diagnosis and vaccination status relied on self-reporting, which may introduce recall bias and information bias due to variations in diagnostic criteria, potentially compromising the accuracy of the results. Moreover, key covariates such as cardiovascular medication use and prior history of COVID-19 infection were not included, and detailed information on the timing of vaccination was lacking. Additionally, since 94.3% of participants received mRNA vaccines, the conclusions may not be generalizable to adenoviral vector vaccines, given their differing immune responses and risks of myocarditis.

Therefore, future work will involve validating CHD diagnoses using clinical records or other objective measures to minimize self-reporting bias, and verifying the applicability of our results across diverse populations through international multi-center collaborations that integrate cohorts from Europe, Asia, and other regions. Concurrently, we plan to prospectively collect cohort data encompassing complete histories of cardiovascular medication use, COVID-19 infection, vaccine types, and other potential confounders. Propensity score matching will be employed to balance baseline differences, thereby reducing potential confounding bias and enabling a deeper exploration of the complex relationship between vaccination and CHD to enhance the robustness and clinical relevance of the findings.

Future mechanistic research should prioritize three axes: (1) serial coronary calcium quantification in pre/post-vaccination CT angiography cohorts; (2) single-cell transcriptomic profiling of plaque macrophages to delineate trained immunity pathways; (3) vaccine platform-stratified analyses leveraging emerging global surveillance datasets. Population-level modeling suggests that if a causal link is established between vaccine-adjuvant cardioprotection and reduced coronary heart disease (CHD) risk, integrating these findings into primary prevention frameworks could theoretically avert approximately 1.2 million CHD cases annually in high-income countries. Nevertheless, this remains speculative, underscoring the urgency of translating vaccine-adjuvant cardioprotection from the laboratory to population-level implementation.

## Data Availability

The original contributions presented in the study are included in the article/Supplementary material, further inquiries can be directed to the corresponding authors.

## Footnotes

^1^ https://www.cdc.gov/nchs/nhis/index.html

## Glossary

CHD: Coronary Heart Disease
NHIS: National Health Interview Survey
OR: Odds Ratio
CI: Confidence Interval
BMI: Body Mass Index
AUC: Area Under the Curve
ROC: Receiver Operating Characteristic
WHO: World Health Organization
LDL: Low-Density Lipoprotein
MACE: Major Adverse Cardiovascular Events
ACE2: Angiotensin-Converting Enzyme 2
IL-6: Interleukin-6
TNF-α: Tumor Necrosis Factor-alpha
NLRP3: NLR Family Pyrin Domain Containing 3
MMP: Matrix Metalloproteinase
PCSK9: Proprotein Convertase Subtilisin/Kexin Type 9
HDAC2: Histone Deacetylase 2
nAChR: Nicotinic Acetylcholine Receptor
ACS: Acute Coronary Syndrome
CT: Computed Tomography
CDC: Centers for Disease Control and Prevention
mRNA: Messenger RNA
