# Supplementary material for: Association between COVID-19 vaccination and coronary heart disease: based on 2023 national health interview survey data
Source: Front Public Health. 2025 Sep 15;13:1641156. doi: 10.3389/fpubh.2025.1641156 (PMC12478238; doi:10.3389/fpubh.2025.1641156)
Supplement: Supplementary file 1 [file Supplementary_file_1.docx]

Supplementary Material

## 1 Supplementary Figures


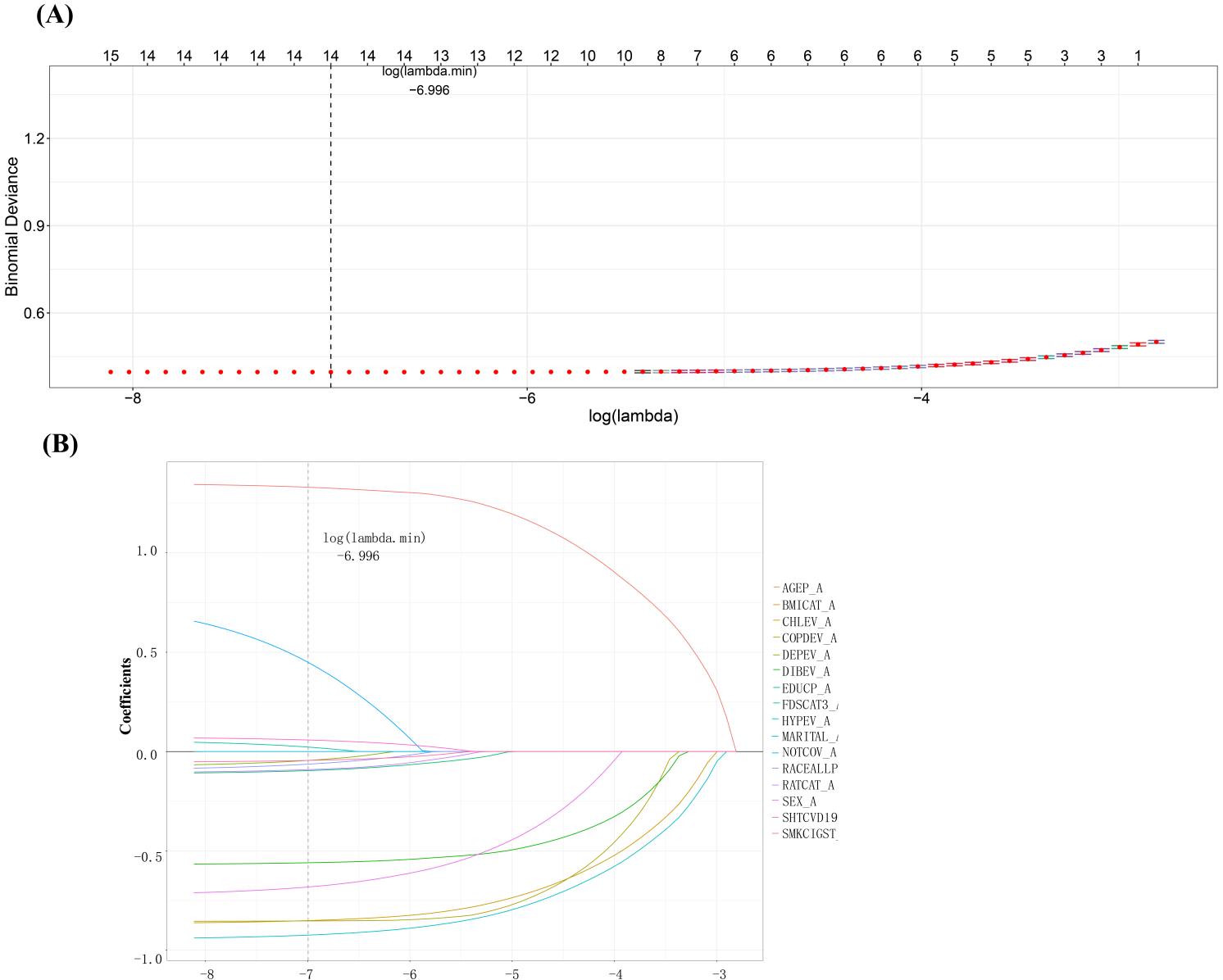


**Supplementary Figure 1.** LASSO regression analysis was performed for screening feature variables.

**(A)** Partial likelihood deviance curve. log(lambda.min) = -6.996 corresponds to the log value of the optimal regularization parameter λ, which was determined based on the "minimum partial likelihood deviance" criterion. **(B)** LASSO regression coefficient profile plot. Variables with non-zero coefficients at log(lambda.min) = -6.996 were screened as feature indices (14 variables retained in the end).

1. **Supplementary Tables**

**Supplementary Table 1.** Table of stepwise sample exclusion

| Exclusion conditions | Identifier | Exclude | Number of samples | Proportion |
| --- | --- | --- | --- | --- |
| All |  |  | 29522 |  |
| age<18 and no information | AGEP_A | 65 | 29457 | 0.22% |
| without sex information | SEX_A | 6 | 29451 | 0.02% |
| Race samples without information | RACEALLP_A | 1597 | 27854 | 5.41% |
| Marital marriage without information | MARITAL_A | 1116 | 26738 | 4.17% |
| No information on education level | EDUCP_A | 102 | 26636 | 0.49% |
| RATCAT poverty status | RATCAT_A | 0 | 26636 | 0.00% |
| Without health insurance information | NOTCOV_A | 55 | 26581 | 0.31% |
| Smoking status | SMKCIGST_A | 57 | 26524 | 3.53% |
| Samples without BMI information | BMICAT_A | 480 | 26044 | 2.13% |
| Food safety | FDSCAT3_A | 146 | 25898 | 4.63% |
| Diabetes | DIBEV_A | 14 | 25884 | 0.11% |
| Hypertension | HYPEV_A | 15 | 25869 | 0.17% |
| High cholesterol | CHLEV_A | 44 | 25825 | 0.29% |
| Depression | DEPEV_A | 20 | 25805 | 0.15% |
| Lung disease | COPDEV_A | 11 | 25794 | 0.14% |
| Coronary heart disease | CHDEV_A | 55 | 25739 | 0.31% |
| SHTCVD19NM1_A | SHTCVD19NM1_A | 4833 | 20906 | 20.59% |

**Supplementary Table 2.** Table of variance inflation factors between variables in Model 3

|  | GVIF | Df | GVIF^(1/(2*Df)) |
| --- | --- | --- | --- |
| SHTCVD19NM1_A | 1.113 | 5 | 1.011 |
| AGEP_A | 1.207 | 1 | 1.099 |
| SEX_A | 1.106 | 1 | 1.052 |
| RACEALLP_A | 1.147 | 5 | 1.014 |
| MARITAL_A | 1.166 | 2 | 1.039 |
| EDUCP_A | 1.221 | 2 | 1.051 |
| RATCAT_A | 1.368 | 2 | 1.081 |
| NOTCOV_A | 1.025 | 1 | 1.013 |
| SMKCIGST_A | 1.212 | 3 | 1.033 |
| BMICAT_A | 1.151 | 3 | 1.024 |
| FDSCAT3_A | 1.173 | 2 | 1.041 |
| DIBEV_A | 1.121 | 1 | 1.059 |
| HYPEV_A | 1.168 | 1 | 1.081 |
| DEPEV_A | 1.111 | 1 | 1.054 |
| CHLEV_A | 1.113 | 1 | 1.055 |
| COPDEV_A | 1.147 | 1 | 1.071 |

Abbreviations: VIF: Variance inflation factors; OR: Odds ratio.

**Supplementary Table 3.** The association between covid-19 vaccination and coronary heart disease in new model 3

| Exposure | | Variable | Model 3 | |
| --- | --- | --- | --- | --- |
|  |  |  | OR(95%CI) | P-value |
| 1 | SHTCVD19NM1_A | Ref. | Ref. | Ref. |
| 2 | SHTCVD19NM1_A | SHTCVD19NM1_A2 | 0.686(0.483~0.953) | 0.029 |
| 3 | SHTCVD19NM1_A | SHTCVD19NM1_A3 | 0.623(0.441~0.861) | 0.006 |
| 4 | SHTCVD19NM1_A | SHTCVD19NM1_A4 | 0.637(0.448~0.886) | 0.009 |
| 5 | SHTCVD19NM1_A | SHTCVD19NM1_A5 | 0.610(0.419~0.871) | 0.008 |
| 6 | SHTCVD19NM1_A | SHTCVD19NM1_A6 | 0.457(0.288~0.724) | 0.001 |

Model 3: Incorporating 14 variables, AGEP_A, SEX_A, RACEALLP_A, EDUCP_A, RATCAT_A, NOTCOV_A, SMKCIGST_A, FDSCAT3_A, DIBEV_A, HYPEV_A, DEPEV_A, CHLEV_A, COPDEV_A, SHTCVD19NM1_A.

Statistical metrics: Odds ratios (ORs) and 95% confidence intervals (CIs) are reported. Reference category: 1 vaccination.

Key findings: Dose-response trend: Increasing vaccinations associated with reduced CHD risk.

Significance: All P values <0.05 indicate statistical significance.

Abbreviations: CI: Confidence interval; OR: Odds ratio.
